# 6-(1-Adamant­yl)-3-(2-fluoro­phen­yl)-1,2,4-triazolo[3,4-*b*][1,3,4]thia­diazole

**DOI:** 10.1107/S1600536809019692

**Published:** 2009-05-29

**Authors:** Mahmood-ul-Hassan Khan, Shahid Hameed, M. Nawaz Tahir, Tanveer Hussain Bokhari, Islam Ullah Khan

**Affiliations:** a Department of Chemistry, Quaid-i-Azam University, Islamabad 45320, Pakistan; b Department of Physics, University of Sargodha, Sargodha, Pakistan; c Department of Chemistry, Government College University, Lahore, Pakistan

## Abstract

In the title compound, C_19_H_19_FN_4_S, the planes of the 2-fluoro­phenyl and 1,2,4-triazolo[3,4-*b*][1,3,4]thia­diazole ring systems are oriented at a dihedral angle of 48.98 (6)°. In the crystal, weak C—H⋯S and C—H⋯π inter­actions may help to establish the packing and π–π inter­actions between the centroids of the benzene rings at a distance of 3.8792 (13) Å occur.

## Related literature

For a related structure, see: Holm *et al.* (2008 ▶). For our previous studies on related compounds, see: Akhtar *et al.* (2007 ▶, 2008*a* ▶,*b* ▶). For background to the biological activity of related compounds, see: El-Emam *et al.* (2004 ▶); Kadi *et al.* (2007 ▶); Zhang *et al.* (2002 ▶).
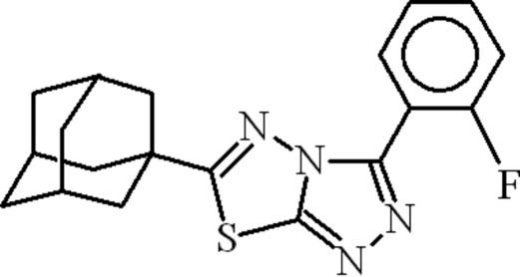


## Experimental

### 

#### Crystal data

C_19_H_19_FN_4_S
*M* *_r_* = 354.44
Orthorhombic, 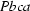

*a* = 13.2684 (9) Å
*b* = 12.4293 (9) Å
*c* = 20.2231 (15) Å
*V* = 3335.1 (4) Å^3^
*Z* = 8
Mo *K*α radiation
μ = 0.21 mm^−1^
*T* = 296 K
0.25 × 0.22 × 0.20 mm

#### Data collection

Bruker Kappa APEXII CCD diffractometer
Absorption correction: multi-scan (*SADABS*; Bruker, 2005 ▶) *T*_min_ = 0.945, *T*_max_ = 0.956
22695 measured reflections
4486 independent reflections
3116 reflections with *I* > 2σ(*I*)
*R*_int_ = 0.031

#### Refinement

*R*[*F* ^2^ > 2σ(*F* ^2^)] = 0.047
*wR*(*F* ^2^) = 0.136
*S* = 1.04
4486 reflections
235 parameters
H-atom parameters constrained
Δρ_max_ = 0.35 e Å^−3^
Δρ_min_ = −0.28 e Å^−3^

### 

Data collection: *APEX2* (Bruker, 2007 ▶); cell refinement: *SAINT* (Bruker, 2007 ▶); data reduction: *SAINT*; program(s) used to solve structure: *SHELXS97* (Sheldrick, 2008 ▶); program(s) used to refine structure: *SHELXL97* (Sheldrick, 2008 ▶); molecular graphics: *ORTEP-3* (Farrugia, 1997 ▶) and *PLATON* (Spek, 2009 ▶); software used to prepare material for publication: *WinGX* (Farrugia, 1999 ▶) and *PLATON*.

## Supplementary Material

Crystal structure: contains datablocks global, I. DOI: 10.1107/S1600536809019692/hb2984sup1.cif

Structure factors: contains datablocks I. DOI: 10.1107/S1600536809019692/hb2984Isup2.hkl

Additional supplementary materials:  crystallographic information; 3D view; checkCIF report

## Figures and Tables

**Table 1:** Hydrogen-bond geometry (Å, °)

*D*—H⋯*A*	*D*—H	H⋯*A*	*D*⋯*A*	*D*—H⋯*A*
C15—H15*A*⋯S1^i^	0.97	2.84	3.640 (2)	140
C12—H12⋯*Cg*1^ii^	0.98	2.90	3.713 (2)	140
C18—H18*B*⋯*Cg*1^iii^	0.97	2.83	3.787 (3)	170

Symmetry codes: (i) 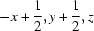; (ii) 
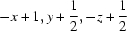
; (iii) 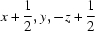. *Cg*1 is the centroid of the S1/C8/N3/N4/C9 ring.

**Fig. 1.**
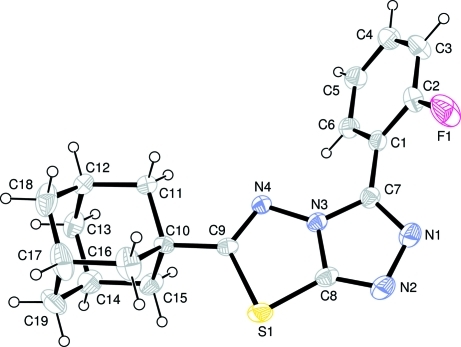
View of (I) with displacement ellipsoids drawn at the 30% probability level. H-atoms are shown by small spheres of arbitrary radius.

**Fig. 2.**
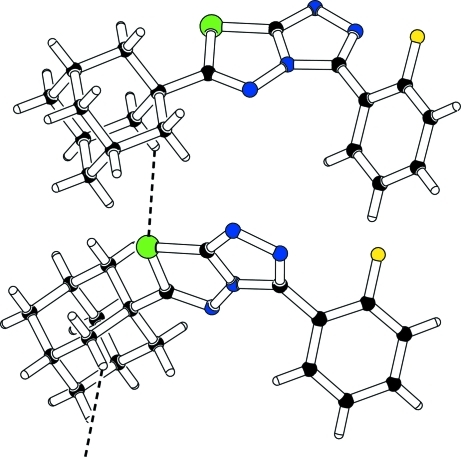
Partial packing diagram for (I) showing the C—H···S interaction as a dashed line.

## supplementary crystallographic information

### Comment

Five membered heterocycles bearing adamantyl moiety are gaining importance due to their promising biological activities (El-Emam *et al.*, 2004; Kadi *et al.*, 2007). On the other hand the condensed heterocyclic systems *e.g.*, triazolothiadiazoles exhibit numerous biological activities (Zhang *et al.*, 2002). In continuation of our previous studeis (Akhtar *et al.*, 2007, 2008*a*, 2008*b*), the title compound, (I), (Fig. 1), has been synthesized with the hope that it will possess antiviral and anticancer activity. The crystal structure of (II) 3-(2-Fluorophenyl)-6-(phenoxymethyl)-1,2,4- triazolo(3,4 - b)(1,3,4)thiadiazole (Holm, *et al.*, 2008) has been published which has a common moiety as in (I).

In (I), the ring A (C1—C6) along with F1-atom and two [(C7/N1/N2/C8/N3) & (C8/S1/C9/N4/N3)] fused heterocyclic rings B (C7/N1/N2/C8/S1/C9/N4/N3) are planar and oriented at a dihedral angle of 48.98 (6)°. There exist π—π interaction between the centroids, CgA—CgA^i^ [symmetry code: i = 1 - *x*, -*y*, 1 - *z*], of benzene rings at a distance of 3.8792 (13) Å. In the crystal, the packing is consolidated by H-bonding (Fig 2) and C—H···π interactions (Table 1).

### Experimental

A mixture of 4-amino-5-(2-fluorophenyl)-2*H*-1,2,4-triazole-3(4*H*)-thione (0.2 g, 0.56 mmol) and adamantane-1-carboxylic acid (0.10 g, 0.56 mmol) in the presence of POCl_3_ (5 ml) was refluxed for four hours. The reaction mixture was cooled to room temperature, poured into crushed ice and neutralized using solid potassium carbonate until pH was 8. The precipitated solid was filtered, washed with excess water and recrystallized from chloroform to yield colourless prisms of (I).

### Refinement

H-atoms were positioned in calculated positions with C-H = 0.93–0.98 Å and refined as riding with U_iso_(H) = 1.2U_eq_(C).

### Crystal data

**Table:**

C_19_H_19_FN_4_S	*F*(000) = 1488
*M**_r_* = 354.44	*D*_x_ = 1.412 Mg m^−^^3^
Orthorhombic, *P**b**c**a*	Mo *K*α radiation, λ = 0.71073 Å
Hall symbol: -P 2ac 2ab	Cell parameters from 3116 reflections
*a* = 13.2684 (9) Å	θ = 2.5–29.1°
*b* = 12.4293 (9) Å	µ = 0.21 mm^−^^1^
*c* = 20.2231 (15) Å	*T* = 296 K
*V* = 3335.1 (4) Å^3^	Prism, colourless
*Z* = 8	0.25 × 0.22 × 0.20 mm

### Data collection

**Table:**

Bruker Kappa APEXII CCD diffractometer	4486 independent reflections
Radiation source: fine-focus sealed tube	3116 reflections with *I* > 2σ(*I*)
graphite	*R*_int_ = 0.031
Detector resolution: 7.40 pixels mm^-1^	θ_max_ = 29.1°, θ_min_ = 2.5°
ω scans	*h* = −12→18
Absorption correction: multi-scan (*SADABS*; Bruker, 2005)	*k* = −17→14
*T*_min_ = 0.945, *T*_max_ = 0.956	*l* = −26→27
22695 measured reflections	

### Refinement

**Table:**

Refinement on *F*^2^	Primary atom site location: structure-invariant direct methods
Least-squares matrix: full	Secondary atom site location: difference Fourier map
*R*[*F*^2^ > 2σ(*F*^2^)] = 0.047	Hydrogen site location: inferred from neighbouring sites
*wR*(*F*^2^) = 0.136	H-atom parameters constrained
*S* = 1.04	*w* = 1/[σ^2^(*F*_o_^2^) + (0.0579*P*)^2^ + 1.3259*P*] where *P* = (*F*_o_^2^ + 2*F*_c_^2^)/3
4486 reflections	(Δ/σ)_max_ < 0.001
235 parameters	Δρ_max_ = 0.35 e Å^−^^3^
0 restraints	Δρ_min_ = −0.28 e Å^−^^3^

### Special details

**Table:**

Geometry. Bond distances, angles *etc*. have been calculated using the rounded fractional coordinates. All su's are estimated from the variances of the (full) variance-covariance matrix. The cell e.s.d.'s are taken into account in the estimation of distances, angles and torsion angles
Refinement. Refinement of *F*^2^ against ALL reflections. The weighted *R*-factor *wR* and goodness of fit *S* are based on *F*^2^, conventional *R*-factors *R* are based on *F*, with *F* set to zero for negative *F*^2^. The threshold expression of *F*^2^ > σ(*F*^2^) is used only for calculating *R*-factors(gt) *etc*. and is not relevant to the choice of reflections for refinement. *R*-factors based on *F*^2^ are statistically about twice as large as those based on *F*, and *R*- factors based on ALL data will be even larger.

### Fractional atomic coordinates and isotropic or equivalent isotropic displacement parameters (Å^2^)

**Table:**

	*x*	*y*	*z*	*U*_iso_*/*U*_eq_	
S1	0.25232 (4)	−0.15058 (4)	0.23126 (2)	0.0517 (2)	
F1	0.35571 (14)	−0.27396 (11)	0.52074 (7)	0.0839 (6)	
N1	0.20243 (13)	−0.22822 (13)	0.41446 (9)	0.0543 (6)	
N2	0.17459 (14)	−0.24778 (14)	0.34844 (9)	0.0591 (6)	
N3	0.29443 (10)	−0.12526 (11)	0.35182 (7)	0.0360 (4)	
N4	0.36117 (10)	−0.05549 (11)	0.32199 (7)	0.0343 (4)	
C1	0.32252 (13)	−0.10525 (14)	0.47314 (9)	0.0392 (5)	
C2	0.36251 (16)	−0.16551 (17)	0.52410 (10)	0.0517 (6)	
C3	0.41073 (19)	−0.1200 (2)	0.57717 (10)	0.0639 (8)	
C4	0.41784 (17)	−0.0094 (2)	0.58048 (10)	0.0611 (8)	
C5	0.37749 (16)	0.05301 (18)	0.53119 (10)	0.0549 (7)	
C6	0.33120 (14)	0.00584 (15)	0.47758 (9)	0.0463 (6)	
C7	0.27374 (13)	−0.15494 (14)	0.41576 (9)	0.0403 (5)	
C8	0.23167 (13)	−0.18403 (14)	0.31343 (10)	0.0437 (5)	
C9	0.34740 (12)	−0.06062 (13)	0.25887 (8)	0.0348 (5)	
C10	0.40431 (12)	0.00535 (13)	0.20943 (8)	0.0337 (4)	
C11	0.48759 (15)	0.07039 (18)	0.24376 (9)	0.0504 (6)	
C12	0.54359 (15)	0.13979 (18)	0.19241 (10)	0.0543 (7)	
C13	0.47109 (16)	0.21626 (17)	0.15982 (11)	0.0581 (7)	
C14	0.38813 (17)	0.15387 (18)	0.12638 (12)	0.0626 (8)	
C15	0.33138 (15)	0.08502 (16)	0.17677 (11)	0.0527 (6)	
C16	0.4512 (2)	−0.06639 (17)	0.15637 (11)	0.0657 (8)	
C17	0.5082 (3)	0.0039 (2)	0.10590 (12)	0.0802 (9)	
C18	0.59042 (18)	0.0674 (2)	0.14102 (13)	0.0730 (9)	
C19	0.4329 (3)	0.0822 (2)	0.07399 (11)	0.0855 (10)	
H3	0.43812	−0.16293	0.61028	0.0767*	
H4	0.45004	0.02286	0.61619	0.0733*	
H5	0.38143	0.12757	0.53400	0.0658*	
H6	0.30545	0.04888	0.44397	0.0555*	
H11A	0.45818	0.11629	0.27742	0.0604*	
H11B	0.53468	0.02185	0.26512	0.0604*	
H12	0.59673	0.18097	0.21460	0.0651*	
H13A	0.44245	0.26409	0.19273	0.0697*	
H13B	0.50652	0.25966	0.12744	0.0697*	
H14	0.34099	0.20433	0.10564	0.0751*	
H15A	0.30145	0.13091	0.21022	0.0633*	
H15B	0.27764	0.04595	0.15483	0.0633*	
H16A	0.49735	−0.11705	0.17670	0.0789*	
H16B	0.39877	−0.10692	0.13405	0.0789*	
H17	0.53836	−0.04191	0.07176	0.0963*	
H18A	0.62742	0.11032	0.10915	0.0876*	
H18B	0.63734	0.01826	0.16203	0.0876*	
H19A	0.37972	0.04208	0.05216	0.1027*	
H19B	0.46694	0.12570	0.04103	0.1027*	

### Atomic displacement parameters (Å^2^)

**Table:**

	*U*^11^	*U*^22^	*U*^33^	*U*^12^	*U*^13^	*U*^23^
S1	0.0578 (3)	0.0481 (3)	0.0492 (3)	−0.0163 (2)	−0.0141 (2)	−0.0060 (2)
F1	0.1293 (14)	0.0492 (8)	0.0733 (9)	0.0040 (8)	−0.0198 (9)	0.0189 (7)
N1	0.0552 (10)	0.0455 (9)	0.0623 (11)	−0.0141 (8)	−0.0018 (8)	0.0070 (8)
N2	0.0611 (10)	0.0483 (9)	0.0678 (12)	−0.0202 (8)	−0.0068 (9)	0.0020 (9)
N3	0.0342 (7)	0.0312 (7)	0.0427 (8)	−0.0025 (5)	−0.0031 (6)	−0.0002 (6)
N4	0.0330 (7)	0.0324 (7)	0.0375 (7)	−0.0016 (5)	−0.0019 (6)	−0.0014 (6)
C1	0.0351 (8)	0.0425 (9)	0.0401 (9)	−0.0011 (7)	0.0052 (7)	0.0042 (7)
C2	0.0615 (12)	0.0487 (11)	0.0450 (10)	0.0004 (9)	0.0043 (9)	0.0110 (9)
C3	0.0733 (15)	0.0777 (16)	0.0407 (10)	0.0016 (12)	−0.0069 (10)	0.0148 (10)
C4	0.0616 (13)	0.0810 (16)	0.0406 (10)	−0.0128 (12)	0.0007 (9)	−0.0047 (10)
C5	0.0585 (12)	0.0528 (12)	0.0533 (12)	−0.0067 (10)	0.0026 (10)	−0.0077 (9)
C6	0.0470 (10)	0.0432 (10)	0.0487 (10)	−0.0015 (8)	−0.0022 (8)	0.0024 (8)
C7	0.0390 (9)	0.0342 (8)	0.0478 (10)	−0.0005 (7)	0.0006 (7)	0.0048 (7)
C8	0.0425 (9)	0.0356 (9)	0.0530 (10)	−0.0064 (7)	−0.0083 (8)	−0.0036 (8)
C9	0.0338 (8)	0.0313 (7)	0.0394 (9)	0.0013 (6)	−0.0055 (7)	−0.0054 (7)
C10	0.0355 (8)	0.0340 (8)	0.0315 (7)	0.0043 (6)	−0.0038 (6)	−0.0048 (6)
C11	0.0423 (10)	0.0682 (13)	0.0406 (9)	−0.0147 (9)	−0.0045 (8)	0.0014 (9)
C12	0.0401 (10)	0.0728 (14)	0.0499 (11)	−0.0137 (9)	−0.0001 (8)	0.0052 (10)
C13	0.0550 (12)	0.0478 (11)	0.0716 (14)	−0.0041 (9)	0.0134 (11)	0.0044 (10)
C14	0.0527 (12)	0.0602 (13)	0.0748 (15)	0.0024 (10)	−0.0127 (11)	0.0291 (12)
C15	0.0402 (10)	0.0453 (10)	0.0727 (13)	0.0009 (8)	−0.0095 (9)	0.0140 (10)
C16	0.0922 (18)	0.0451 (11)	0.0599 (13)	0.0105 (11)	0.0243 (12)	−0.0122 (10)
C17	0.123 (2)	0.0619 (15)	0.0558 (13)	0.0071 (15)	0.0433 (15)	−0.0135 (12)
C18	0.0537 (13)	0.0819 (17)	0.0834 (17)	0.0217 (12)	0.0285 (12)	0.0241 (14)
C19	0.114 (2)	0.105 (2)	0.0374 (11)	−0.0375 (19)	−0.0114 (13)	0.0115 (13)

### Geometric parameters (Å, °)

**Table:**

S1—C8	1.735 (2)	C14—C15	1.529 (3)
S1—C9	1.7758 (17)	C14—C19	1.506 (4)
F1—C2	1.353 (3)	C16—C17	1.542 (4)
N1—N2	1.407 (3)	C17—C18	1.522 (4)
N1—C7	1.314 (2)	C17—C19	1.537 (5)
N2—C8	1.305 (3)	C3—H3	0.9300
N3—N4	1.3784 (19)	C4—H4	0.9300
N3—C7	1.372 (2)	C5—H5	0.9300
N3—C8	1.353 (2)	C6—H6	0.9300
N4—C9	1.291 (2)	C11—H11A	0.9700
C1—C2	1.380 (3)	C11—H11B	0.9700
C1—C6	1.389 (3)	C12—H12	0.9800
C1—C7	1.465 (3)	C13—H13A	0.9700
C2—C3	1.372 (3)	C13—H13B	0.9700
C3—C4	1.380 (4)	C14—H14	0.9800
C4—C5	1.372 (3)	C15—H15A	0.9700
C5—C6	1.377 (3)	C15—H15B	0.9700
C9—C10	1.497 (2)	C16—H16A	0.9700
C10—C11	1.535 (3)	C16—H16B	0.9700
C10—C15	1.534 (3)	C17—H17	0.9800
C10—C16	1.528 (3)	C18—H18A	0.9700
C11—C12	1.541 (3)	C18—H18B	0.9700
C12—C13	1.504 (3)	C19—H19A	0.9700
C12—C18	1.509 (3)	C19—H19B	0.9700
C13—C14	1.507 (3)		
			
C8—S1—C9	87.82 (8)	C2—C3—H3	121.00
N2—N1—C7	109.15 (16)	C4—C3—H3	121.00
N1—N2—C8	104.93 (16)	C3—C4—H4	120.00
N4—N3—C7	135.23 (14)	C5—C4—H4	120.00
N4—N3—C8	118.95 (14)	C4—C5—H5	120.00
C7—N3—C8	105.81 (14)	C6—C5—H5	120.00
N3—N4—C9	108.10 (13)	C1—C6—H6	120.00
C2—C1—C6	117.36 (17)	C5—C6—H6	120.00
C2—C1—C7	122.17 (17)	C10—C11—H11A	110.00
C6—C1—C7	120.47 (16)	C10—C11—H11B	110.00
F1—C2—C1	118.53 (18)	C12—C11—H11A	110.00
F1—C2—C3	118.78 (19)	C12—C11—H11B	110.00
C1—C2—C3	122.7 (2)	H11A—C11—H11B	108.00
C2—C3—C4	118.7 (2)	C11—C12—H12	109.00
C3—C4—C5	120.1 (2)	C13—C12—H12	109.00
C4—C5—C6	120.4 (2)	C18—C12—H12	109.00
C1—C6—C5	120.75 (18)	C12—C13—H13A	110.00
N1—C7—N3	108.16 (16)	C12—C13—H13B	110.00
N1—C7—C1	128.78 (17)	C14—C13—H13A	110.00
N3—C7—C1	122.99 (15)	C14—C13—H13B	110.00
S1—C8—N2	139.20 (16)	H13A—C13—H13B	108.00
S1—C8—N3	108.85 (13)	C13—C14—H14	109.00
N2—C8—N3	111.95 (18)	C15—C14—H14	109.00
S1—C9—N4	116.26 (12)	C19—C14—H14	109.00
S1—C9—C10	119.54 (12)	C10—C15—H15A	110.00
N4—C9—C10	124.18 (15)	C10—C15—H15B	110.00
C9—C10—C11	110.45 (14)	C14—C15—H15A	110.00
C9—C10—C15	108.84 (14)	C14—C15—H15B	110.00
C9—C10—C16	110.78 (14)	H15A—C15—H15B	108.00
C11—C10—C15	107.99 (15)	C10—C16—H16A	110.00
C11—C10—C16	109.38 (16)	C10—C16—H16B	110.00
C15—C10—C16	109.35 (15)	C17—C16—H16A	110.00
C10—C11—C12	109.70 (15)	C17—C16—H16B	110.00
C11—C12—C13	109.91 (17)	H16A—C16—H16B	108.00
C11—C12—C18	109.21 (18)	C16—C17—H17	110.00
C13—C12—C18	109.78 (18)	C18—C17—H17	110.00
C12—C13—C14	109.79 (18)	C19—C17—H17	110.00
C13—C14—C15	110.41 (19)	C12—C18—H18A	110.00
C13—C14—C19	109.4 (2)	C12—C18—H18B	110.00
C15—C14—C19	109.39 (19)	C17—C18—H18A	110.00
C10—C15—C14	109.73 (16)	C17—C18—H18B	110.00
C10—C16—C17	109.51 (17)	H18A—C18—H18B	108.00
C16—C17—C18	109.7 (2)	C14—C19—H19A	110.00
C16—C17—C19	108.5 (3)	C14—C19—H19B	110.00
C18—C17—C19	109.5 (2)	C17—C19—H19A	110.00
C12—C18—C17	109.6 (2)	C17—C19—H19B	110.00
C14—C19—C17	109.61 (19)	H19A—C19—H19B	108.00
			
C9—S1—C8—N2	−179.0 (2)	C4—C5—C6—C1	1.5 (3)
C9—S1—C8—N3	1.05 (13)	S1—C9—C10—C11	−175.71 (12)
C8—S1—C9—N4	−0.64 (14)	S1—C9—C10—C15	65.90 (17)
C8—S1—C9—C10	−179.31 (14)	S1—C9—C10—C16	−54.36 (19)
C7—N1—N2—C8	−0.1 (2)	N4—C9—C10—C11	5.7 (2)
N2—N1—C7—N3	−0.2 (2)	N4—C9—C10—C15	−112.66 (18)
N2—N1—C7—C1	176.70 (17)	N4—C9—C10—C16	127.08 (18)
N1—N2—C8—S1	−179.67 (17)	C9—C10—C11—C12	−178.63 (15)
N1—N2—C8—N3	0.3 (2)	C15—C10—C11—C12	−59.7 (2)
C7—N3—N4—C9	179.60 (18)	C16—C10—C11—C12	59.2 (2)
C8—N3—N4—C9	0.9 (2)	C9—C10—C15—C14	179.60 (16)
N4—N3—C7—N1	−178.48 (16)	C11—C10—C15—C14	59.7 (2)
N4—N3—C7—C1	4.4 (3)	C16—C10—C15—C14	−59.3 (2)
C8—N3—C7—N1	0.33 (19)	C9—C10—C16—C17	179.4 (2)
C8—N3—C7—C1	−176.77 (16)	C11—C10—C16—C17	−58.6 (2)
N4—N3—C8—S1	−1.38 (18)	C15—C10—C16—C17	59.5 (2)
N4—N3—C8—N2	178.67 (15)	C10—C11—C12—C13	60.2 (2)
C7—N3—C8—S1	179.58 (12)	C10—C11—C12—C18	−60.3 (2)
C7—N3—C8—N2	−0.4 (2)	C11—C12—C13—C14	−59.2 (2)
N3—N4—C9—S1	0.02 (17)	C18—C12—C13—C14	61.0 (2)
N3—N4—C9—C10	178.62 (14)	C11—C12—C18—C17	61.0 (2)
C6—C1—C2—F1	−179.52 (18)	C13—C12—C18—C17	−59.6 (2)
C6—C1—C2—C3	−1.0 (3)	C12—C13—C14—C15	59.4 (2)
C7—C1—C2—F1	−0.3 (3)	C12—C13—C14—C19	−61.0 (2)
C7—C1—C2—C3	178.2 (2)	C13—C14—C15—C10	−60.1 (2)
C2—C1—C6—C5	−0.4 (3)	C19—C14—C15—C10	60.3 (2)
C7—C1—C6—C5	−179.61 (18)	C13—C14—C19—C17	59.7 (3)
C2—C1—C7—N1	50.7 (3)	C15—C14—C19—C17	−61.4 (3)
C2—C1—C7—N3	−132.83 (19)	C10—C16—C17—C18	59.5 (3)
C6—C1—C7—N1	−130.1 (2)	C10—C16—C17—C19	−60.1 (2)
C6—C1—C7—N3	46.3 (3)	C16—C17—C18—C12	−60.8 (3)
F1—C2—C3—C4	179.9 (2)	C19—C17—C18—C12	58.2 (3)
C1—C2—C3—C4	1.3 (3)	C16—C17—C19—C14	61.2 (3)
C2—C3—C4—C5	−0.3 (3)	C18—C17—C19—C14	−58.5 (3)
C3—C4—C5—C6	−1.1 (3)		

### Hydrogen-bond geometry (Å, °)

**Table:**

*D*—H···*A*	*D*—H	H···*A*	*D*···*A*	*D*—H···*A*
C15—H15A···S1^i^	0.97	2.84	3.640 (2)	140
C12—H12···Cg1^ii^	0.98	2.90	3.713 (2)	140
C18—H18B···Cg1^iii^	0.97	2.83	3.787 (3)	170

Symmetry codes: (i) −*x*+1/2, *y*+1/2, *z*; (ii) −*x*+1, *y*+1/2, −*z*+1/2; (iii) *x*+1/2, *y*, −*z*+1/2.
